# The impact of self-modeling on athletic performance and self-efficacy: a systematic review and meta-analysis

**DOI:** 10.3389/fspor.2026.1809566

**Published:** 2026-08-19

**Authors:** Ziyi Wang, Haobai Li, Qi Wang, Zhikun Gao, Jianfeng Niu

**Affiliations:** 1 Sports Coaching College, Beijing Sport University, Beijing, China; 2 Physical Education Department, Beijing International Studies University, Beijing, China; 3 Humanistic Quality Education Center, Beijing Youth Politics College, Beijing, China

**Keywords:** athletic performance, meta-analysis, self-efficacy, self-modeling, video feedback

## Abstract

**Introduction:**

Self-modeling, as a pedagogical approach in physical education, can effectively promote students' acquisition of movement skills and enhance their self-efficacy. However, the efficacy of self-modeling interventions remains controversial, and there is currently no objective, comprehensive evidence demonstrating their effectiveness in physical education instruction.

**Methods:**

This study systematically evaluates the effects of self-modeling as an observational learning intervention on athletic performance and self-efficacy, along with its operational conditions, through a systematic review and meta-analysis.

**Results:**

Self-modeling yields moderate to large positive influences on athletic performance and significantly enhances self-efficacy to a moderate degree. One week after the intervention ended, however, the improvement in athletic performance did not persist. Regression analysis revealed that intervention dose was the primary factor influencing effectiveness, with larger doses yielding more pronounced effects. Age, exercise experience, skill classification, and control group intervention type did not demonstrate significant moderating effects.

**Discussion:**

The use of self-modeling in physical education and competitive sports can effectively enhance athletic performance and self-efficacy. However, its effects are dose-dependent and exhibit limited short-term sustainability. Future research should further clarify optimal intervention protocols and long-term mechanisms.

**Systematic Review Registration:**

https://www.crd.york.ac.uk/prospero/display_record.php?ID=CRD420261477126, PROSPERO CRD420261477126.

## 1 Introduction

According to Albert Bandura's social cognitive theory, observational learning can positively influence athletic performance and cognition. In observational learning, individuals observe model behaviors within a given environment, which subsequently influences their own information processing and potentially impacts personal behavior (1). Self-observation, as a form of observational learning, assists observers in rapidly acquiring essential information about their own performance and outcomes (2). Viewing video recordings of one's own actions is a common method of self-observation. It should be noted, however, that the degree to which self-observation is implemented should be commensurate with the observer's own skill level (3, 4).

Self-modeling is an advanced form of self-observation, in which individuals consolidate their successful experiences by editing and replaying highlights of their best performances. It even involves creating edited versions that surpass their current level, thereby shaping an ideal self-image. This intervention aligns with Barry J. Zimmerman's self-regulation model, utilizing video editing to reveal observers' “higher-level self-performance,” thereby providing positive internal reinforcement (5, 6). When observers perceive that they can perform better, they often develop a belief in their own potential for improvement, thereby enhancing their self-efficacy. Enhanced self-efficacy subsequently stimulates intrinsic motivation, strengthens persistence, and encourages the setting of challenging goals, thereby indirectly promoting skill acquisition. During this process, observers contrast their actual performance with the exemplary demonstration in the video, fostering positive causal attributions (7). Such attributions further strengthen cognitive confidence in subsequent learning, creating a reinforcing cycle of cognition–performance–cognition (8, 9). Theoretically, this effect is typically more pronounced than observing others, as the self-model is more closely linked to the observer's own motor experience, resulting in a more direct reinforcing effect. In recent years, self-modeling has received widespread attention in the fields of sports psychology and skill learning. Its function extends beyond enhancing athletic performance to include the regulation of cognitive and emotional processes (1, 6). Research indicates that self-modeling not only effectively promotes the learning and execution of motor skills but may also enhance observers' self-regulation abilities, such as increasing self-efficacy (10), intrinsic motivation, and satisfaction with tasks (11). Particularly when learning complex or continuous motor tasks, self-modeling helps observers establish positive psychological expectations and technical structures by presenting potential future performance, thereby generating positive effects at both cognitive and emotional levels (12). In addition, this approach is also applicable in competitive settings, enabling athletes to adjust their arousal levels, enhance focus, reduce competitive anxiety, and facilitate the automatic execution of technical strategies through repeated precompetition viewing of videos of successful performances (12, 13). However, existing research has largely focused on skill learning in training contexts, and there remains a lack of systematic evidence regarding the impact of self-modeling on competitive performance in formal competitions.

Although self-modeling has demonstrated positive effects in multiple studies, controversy persists in this field (14). For example, research indicates that in learning bicycle start skills, the self-modeling group and the traditional verbal feedback group showed insignificant differences in skill performance (15). A recent study also found that in children's freestyle swimming instruction, there was no significant difference in performance improvement among the three groups—self-modeling, self-observation, and peer modeling (16). These inconsistent findings suggest that the advantages of self-modeling are not universally present or remain dependent on specific conditions. Furthermore, the causal pathways of self-modeling remain unclear. Some studies suggest that self-modeling indirectly promotes skill acquisition by enhancing learners' self-regulation processes, such as self-efficacy and intrinsic motivation (11). Some research also indicates that the positive effects of self-modeling may primarily stem from direct information processing and attention-directing mechanisms, without significant changes in psychological variables such as motivation or self-efficacy (12, 16, 17). Finally, factors such as task attributes (e.g., whether skills are sequential or serial), learner age, and initial skill level are often considered potential moderating variables. In particular, the existing evidence on how these factors moderate self-efficacy outcomes remains fragmented and inconclusive (18, 19), leaving the conditions under which self-modeling is applicable and the most effective implementation scenarios yet to be clearly defined (3, 20).

In summary, to systematically clarify the validity and conditions for the effectiveness of self-modeling, and to analyze its underlying mechanisms, this study synthesizes existing research on the effects of self-modeling on athletic performance and self-efficacy, and conducts a meta-analysis of the included data to determine its effectiveness. In addition, through meta-regression analysis and subgroup analyses, we further explore the moderating effects of key factors such as skill classification, age, and intervention dosage on the effectiveness of self-modeling. This study seeks to provide comprehensive conclusions at a higher level of evidence, advance the refinement of observational learning theory, and offer more targeted, scientifically grounded empirical support for sports training and skill instruction practices.

## 2 Material and methods

This systematic review and meta-analysis was conducted in accordance with the Preferred Reporting Items for Systematic Reviews and Meta-Analyses (PRISMA) guidelines (63) and was registered with PROSPERO, an international prospective register of systematic reviews (Registration ID: 1275092).

### 2.1 Data sources and search strategies

Two authors (ZW and HL) independently searched the following electronic databases from inception to 15 September 2025: Web of Science, PubMed, MEDLINE, SPORTDiscus, the Cochrane Library, and PsycINFO. Searches employed Boolean logic (“AND,” “OR”) to combine the following key terms: (“self-modeling” OR “self-referential modeling”) AND (“exercise” OR “sport” OR “workout” OR “physical activity” OR “movement”) OR (“self-efficacy” OR “perceived self-efficacy” OR “self-efficacy belief”). In addition, citation tracing was employed to supplement database searches. This involved manually retrieving reference lists from studies that matched the inclusion criteria. All searches were restricted to English-language publications. Detailed search strategies are provided in Supplementary Table S1.

### 2.2 Selection criteria

All included studies were required to be published peer-reviewed articles. The inclusion criteria followed the PICOS principle: (1) Population: unrestricted; studies were included only if they demonstrated baseline equivalence between the intervention and control groups for all primary outcome measures, with no statistically significant differences at baseline; (2) Intervention: self-modeling administered either as an independent intervention or integrated with other therapies; when combined with additional interventions, control groups were required to exclusively receive those non-self-modeling components to isolate self-modeling-specific effects; (3) Comparison: self-modeling outcomes had to be evaluated against control conditions; (4) Outcomes: the study had to report primary quantitative data assessing motor performance and/or self-efficacy, with sufficient statistical parameters (means, standard deviations, sample sizes) for meta-analysis; and (5) Study design: the study had to adopt randomized controlled trial or randomized crossover controlled trial designs. Articles with the following conditions were excluded: (1) those that did not provide specific data on research results; (2) review papers, conference abstracts, and articles; (3) those with duplicate publications; and (4) non-English publications.

### 2.3 Data extraction

Two authors (ZW and HL) extracted the data independently in accordance with the Cochrane Collaboration Handbook. In cases of disagreement, the data extractors first conducted independent reviews and exchanged views. If consensus could not be reached, the corresponding author (JN) chaired a three-way discussion, using the PICOS inclusion criteria as a basis for judgment and focusing on variables critical to the calculation of effect sizes until consensus was reached. For each study, the mean and standard deviation of the postintervention indicator results were extracted. Once complete, the final author (JN) blind-checked all primary data across the studies and contacted the corresponding authors of studies lacking essential data for the meta-analysis. Studies that did n't respond within 2 weeks or by the cutoff date for data analysis (30 November 2025), whichever was later, were excluded from the final analysis.

### 2.4 Quality assessment

Two authors (ZW and HL) independently assessed the methodological quality of the included studies using the Physiotherapy Evidence Database (PEDro) criteria. The PEDro scale is specifically designed to evaluate the methodological rigor of randomized controlled trials examining physical interventions, making it particularly suitable for appraising the studies in this review (21). The PEDro scale examines critical domains, including randomization, blinding, and allocation concealment, which are essential for safeguarding the internal validity of the studies included in this systematic review and meta-analysis (21).

The PEDro scale comprises 11 items requiring dichotomous (“yes” or “no”) responses. Each “no” response scores 0 points, while each “yes” response scores 1 point. This generates total scores ranging from 0 to 11 per study. Due to the difficulty of implementing blinding, particularly for participants and therapists, in exercise intervention trials (22), the following methodological quality classifications were adapted according to prespecified criteria: scores ≥6 denoted high quality, low risk of bias; scores 4–5 indicated acceptable quality, moderate risk of bias; and scores ≤3 represented low quality, high risk of bias. This study did not set an absolute exclusion threshold based on the PEDro total score, and all randomized controlled trials meeting the PICOS inclusion criteria were included.

Two authors (HL and ZW) independently appraised the quality of the evidence based on the Grading of Recommendations Assessment, Development and Evaluation (GRADE) criteria. These criteria categorize evidence based on study limitations, imprecision, inconsistency, indirectness, and publication bias. If the two authors disagreed on any score, the final author (JN) was consulted until consensus was reached.

### 2.5 Data processing

#### 2.5.1 Effect size calculation

Statistical analyses were performed using the meta (23), metafor (64), tidyverse (24), and dmetar packages (25) in R version 4.5.0 (R Statistical Computing Project). A meta-analysis of the raw data was conducted using the METACONT function in the “meta” package. The METACONT function was chosen because it automatically applies Hedges' correction (26) to Cohen's d estimates from small-sample studies without manual intervention. Although the effect size label displayed in the results may be “standardized mean difference (SMD),” the actual calculation incorporates Hedges' correction (27). Given substantial variations in outcome variable measurement units across studies (e.g., intervention dosage, control methods, populations, and age), the DerSimonian and Laird random-effects model with Hartung–Knapp–Sidik–Jonkman variance adjustment was applied (65). Results were presented with 95% confidence intervals (CIs) and forest plots. We also calculated fixed-effects model results for reference in low-heterogeneity scenarios to enhance result reliability. Effect sizes were categorized as trivial (Hedges' *g* < 0.2), small (0.2 ≤ Hedges’ *g* < 0.5), moderate (0.5 ≤ Hedges' *g* < 0.8), or large (Hedges' *g* ≥ 0.8) (28). Odds ratios were analyzed for categorical outcomes. Heterogeneity was assessed using the *I*^2^ statistic and categorized as low (0%–29%), moderate (30%–49%), substantial (50%–74%), or considerable (75%–100%) (29). Another methodological consideration pertains to the handling of non-independent effect sizes. When multiple effect sizes were derived from the same study, we employed a sample size splitting approach to mitigate the risk of inflating the precision of the pooled estimate. While this is a recognized and pragmatic method, it is a less statistically powerful alternative to more contemporary techniques such as multilevel (hierarchical) meta-analysis or robust variance estimation. These alternative approaches explicitly model the dependency structure among effect sizes and are generally recommended as the preferred method. Our choice was driven by the relatively small number of included studies, which can make more complex models unstable or difficult to fit. Nevertheless, the potential for residual dependency to bias standard errors and heterogeneity estimates (*I*^2^) remains a limitation that should be considered when interpreting the findings.

#### 2.5.2 Data robustness

Sensitivity analyses were conducted using outlier exclusion to demonstrate the robustness of the pooled effect sizes in all comparisons in both meta-analyses. This approach addressed concerns regarding heterogeneity among studies. In particular, the find.outliers function from the R package “dmetar” was used to identify outliers (a study was considered an outlier if its confidence interval did not overlap with the pooled effect's confidence interval) in the primary comparisons of both meta-analyses (27). Outliers were excluded from the pooled effect size estimation. In the exercise and self-efficacy meta-analysis, the specific comparisons included self-modeling versus control conditions.

To mitigate the risk of publication bias, we visually inspected the funnel plot of effect size distributions and subsequently conducted a statistical analysis using Egger's test (30). When the data indicated potential publication bias, we applied the Duval and Tweedie (66) trim-and-fill method to correct the pooled effect estimates at risk of publication bias and determine the true effect size. However, this trimming and filling method may yield invalid results when there is high interstudy heterogeneity (31–33). Therefore, when heterogeneity exceeded 50%, we performed trimming and filling corrections using sensitivity analysis data (i.e., the remaining effect sizes after removing outliers).

### 2.6 Moderator effects

We examined five moderating factors: intervention dose (total time and number of sessions), age, athletic experience, type of control group intervention, and skill classification. We grouped skills as continuous, discrete, or serial. Both dose and age appeared in the two meta-analyses. We analyzed the remaining three—experience, control group type, and skill type—only for motor outcomes. Based on publicly available information from the included studies, we categorized all effect sizes across both meta-analyses according to these moderators. We applied meta-regression to continuous moderators (e.g., intervention dose and age), while we used subgroup analysis for categorical ones (e.g., athletic experience, skill classification, and control group intervention type). In the self-efficacy meta-analysis, we did not run subgroup analyses for the three categorical moderators—control group type, athletic experience, and skill classification. The sample size was too small for statistically reliable comparisons. Each subgroup usually needs at least three studies. Moreover, most studies did not systematically report how these variables interacted with self-efficacy. Without such data, we could not perform meaningful meta-regression.

#### 2.6.1 Intervention dose

The two meta-analyses included in this research encompass both short-term (34, 35) and long-term (15, 16, 36) studies. To achieve a unified assessment, we used total time (in minutes) and the number of sessions as core indicators of intervention dosage. We conducted meta-regression analyses with effect sizes to explore potential associations between intervention dose and outcome levels.

#### 2.6.2 Age

Both meta-analyses in this study utilized age as a moderator variable and performed meta-regression analyses with effect sizes to evaluate the correlation between the effects of age and self-modeling.

#### 2.6.3 Athletic experience

To explore whether athletic experience influences the effectiveness of self-modeling, we examined the presence of athletic experience across two meta-analyses through subgroup analyses.

#### 2.6.4 Skill classification

Subgroup analysis of skill classification is unique to movement meta-analyses. This method uses a one-dimensional approach to categorize primary test skills for movement outcomes (37). The three main approaches to classification are fine versus gross motor tasks, continuous versus discrete motor tasks, and open versus closed tasks. In particular, repetitive movements without a clear beginning or end are categorized as continuous motor tasks (16). Movements with identifiable start and end points are classified as discrete motor tasks (38). A sequence of discrete skills linked together in a specific order to form a skill is categorized as a serial motor task (15). In this analysis, we categorize skills based primarily on continuous, discrete, and serial motor tasks. This is because, in existing research on self-modeling, the debate surrounding continuous and discrete skills is most prominent (16, 39), and this classification system is the most widely used in self-modeling research, facilitating comparisons between research findings.

#### 2.6.5 Control group intervention type

Subgroup analyses by intervention type in the control group are characteristic of motor meta-analyses. Control group interventions are categorized as active or passive. To investigate whether the type of control group intervention influences the effectiveness of self-modeling, we examined the presence of these intervention types in motor meta-analyses using subgroup analyses.

## 3 Results

### 3.1 Study selection

**Figure 1:**
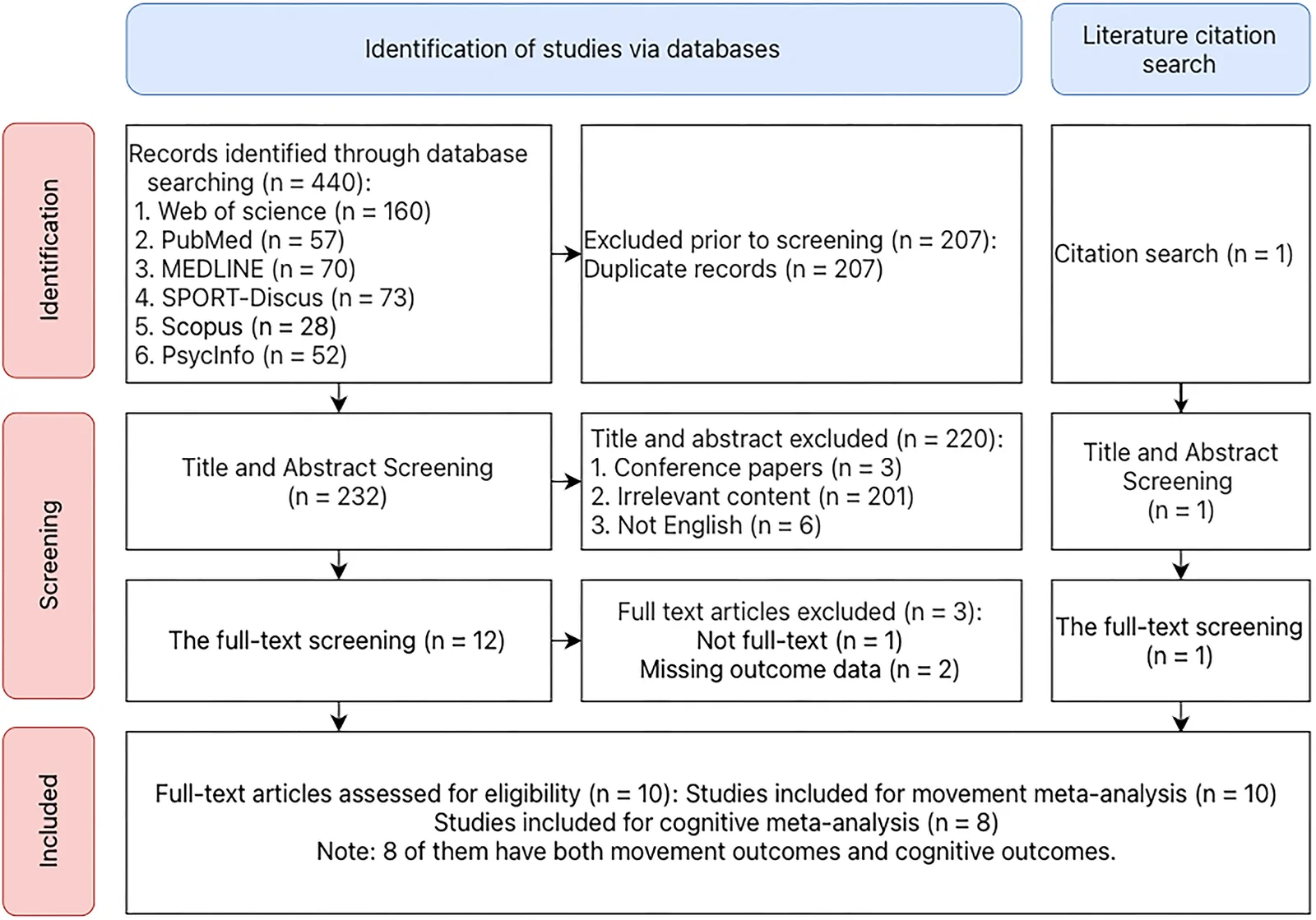
Flow diagram of the study selection process.

The PRISMA flow diagram (Figure 1) illustrates the results of the study screening process. Literature searches were conducted across six databases: Web of Science (*n* = 160), PubMed (*n* = 57), MEDLINE (*n* = 70), SPORT Discus (*n* = 73), Scopus (*n* = 28), and PsycINFO (*n* = 52). A total of 440 publication records were obtained. The initial search period was from September to October 2025. After removing 207 duplicate records, 232 records advanced to the screening phase. During the title and abstract screening phase, 220 records were excluded for the following reasons: three conference papers, 201 irrelevant studies (including those investigating self-observation rather than self-modeling, not measuring motor performance or self-efficacy, or being non-interventional), and six non-English articles. Three additional records were excluded during the full-text screening phase (reasons included: lacking full text: *n* = 1; missing outcome data: *n* = 2). A total of nine studies were ultimately included in the analysis, identified through database searches. One addition study was identified by searching the reference lists of the included studies. After screening titles and abstracts (irrelevant content: *n* = 0) and full texts (missing outcome data: *n* = 0), no records were excluded. Ultimately, one additional study was included via a citation search. The two meta-analyses eventually included a total of 10 studies: the exercise meta-analysis incorporated 10 studies, while the self-efficacy meta-analysis included six studies (which were shared between the exercise and self-efficacy meta-analyses).

### 3.2 Characteristics of included studies

The movement meta-analysis included 26 effect sizes from 10 studies, comprising 16 post-test effect sizes and 10 follow-up effect sizes. The total sample size was 244 participants, including 106 males and 138 females, with an average age of 16.8 years. All participants recruited for this study were healthy individuals. Among the included studies, two employed acute intervention designs, while eight utilized long-term intervention designs. Details are presented in Supplementary Table S2

The self-efficacy meta-analysis included nine effect sizes from six studies. However, due to insufficient follow-up data, these were not analyzed. The total sample size was 137 participants, including 34 males and 103 females, with an average age of 14.47 years. The included studies spanned publication dates from 2005 to 2025. All the participants were healthy, with no history of chronic diseases or sports-related injuries. All studies employed long-term intervention designs. Details are presented in Supplementary Table S3.

### 3.3 Study quality and risk of bias

Quality scores were assigned to the studies included in both meta-analyses using PEDro. The mean quality score for all included studies in the movement meta-analysis was 6.8 ± 0.92, and 90% (9/10) of the studies were classified as high quality (Supplementary Table S4). The quality score for all included studies in the meta-analysis of self-efficacy was 6.67 ± 1.03 on average, with 83.33% (5/6) classified as high-quality studies (Supplementary Table S5). Both meta-analyses identified “allocation concealment,” “subject blinding,” and “provider blinding” as the items that received the lowest ratings. None of the studies included in either analysis met all three conditions. As previously mentioned, this situation arises because of the difficulty in implementing blinding in exercise intervention studies. Overall, all studies demonstrated a high level of rigor.

### 3.4 Movement meta-analysis

**Figure 2:**
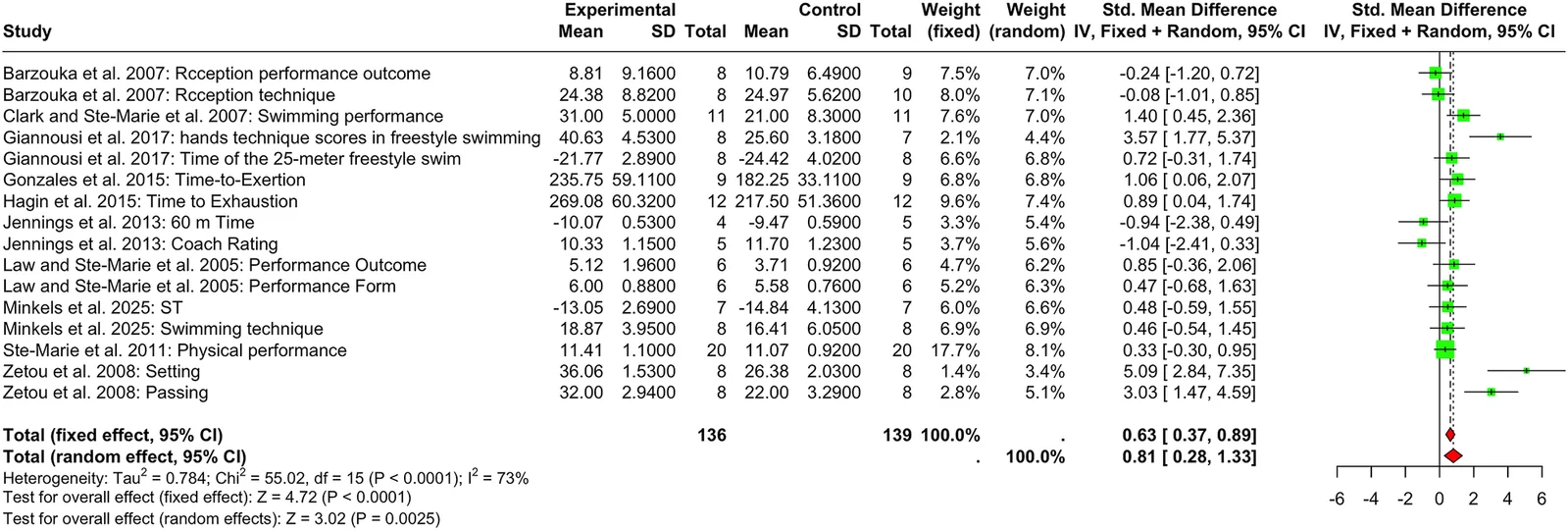
Forest Plot of Effect Sizes Comparing Self-modeling with Control in the Movement Meta-Analysis.

In the movement meta-analysis, 16 effect sizes from 10 studies were included to determine the overall impact of self-modeling on movement outcomes. Compared with the control group, self-modeling had a significantly greater positive overall effect on exercise outcomes [SMD = 0.81, 95% CI (0.28, 1.33), *p* = 0.0025; Figure 2]. Concurrently, there was significantly greater heterogeneity among the studies (*τ*^2^ = 0.784, *I*^2^ = 73%, *p* < 0.01). According to regression analysis, the effect of self-modeling on exercise outcomes was not moderated by age. However, it was moderated by total intervention duration and the number of intervention sessions (Table 1). Subgroup analyses showed that the effect size of the self-modeling intervention was not significantly influenced by athletic experience, skill classification, or control group intervention type (Table 2).

**Table 1:** Meta-regression analyses of the movement outcome data synthesized in the movement meta-analysis.

Subgroup	*N*	*K*	Beta	*P*	Sig.	*τ* ^2^	*I* ^2^
Post-test							
Total time	10	16	0.003	0.0196	Yes	1.44	82.46
Session number	10	16	0.1479	0.0443	Yes	1.34	82.21
Age	10	16	0.0303	0.65	No	1.71	85.42
Follow-up (1 week)							
Total time	5	10	0.0034	0.0004	Yes	0.41	55.11
Session number	5	10	0.23	0.0007	Yes	0.46	58.13
Age	5	10	0.0127	0.94	No	1.8	83.85

*N*, number of studies; *K*,  number of effect sizes; *P*,  significance value; *τ*^2^,  measure of variation around average; *I*^2^, measure of proportion of observed variance (%); Sig. mod., comparison between subgroup categories.

**Table 2:** Subgroup analyses of the movement outcome data synthesized in the movement meta-analysis.

Subgroup	*N*	*K*	SMD	95% CI		*P*	Sig.	*τ* ^2^	*I* ^2^	Sig. mod.
Athletic experience										
Yes	8	12	0.83	0.24	1.43	0.006	Yes	0.76	72	ns
No	2	4	0.79	−0.45	2.03	0.21	No	1.25	80	ref
Skill classification										
Discrete	3	6	1.36	−0.17	3.17	0.16	No	3.56	83	ns
Continuous Serial	5 2	7 3	1.07 −0.35	0.25 −2.36	1.89 1.67	0.02 0.53	Yes No	0.49 0.35	45 59	ref ns
Control group intervention type										
Active	7	13	0.94	−0.08	1.96	0.07	No	2.41	77	ref
Positive	3	3	0.63	−0.35	1.61	0.11	No	0	0	ns

*N*, number of studies; *K*,  number of effect sizes; SMD,  standardized mean difference; 95% CI, lower and upper confidence intervals; *P*,  significance value; *τ*^2^, measure of variation around average; *I*^2^, measure of proportion of observed variance (%); Sig. Mod., comparison between subgroup categories; ref, reference category; ns, no significant difference compared with reference category.

We analyzed 10 follow-up effect sizes from five studies 1 week after the intervention ended to investigate the long-term impact of self-modeling on exercise outcomes. The results indicated that self-modeling did not significantly improve exercise outcomes compared with the control group [SMD = 0.59; 95% CI (−0.09, 1.27); *p* = 0.09]. There was high heterogeneity among the studies (*τ*^2^ = 0.808; *I*^2^ = 70%; *p* < 0.01). Regression analysis indicated that the effect of self-modeling on motor outcomes was not moderated by age, but by total intervention duration and number of intervention sessions (Table 1). Finally, no subgroup analysis was performed due to insufficient follow-up data at other time points.

### 3.5 Self-efficacy meta-analysis

**Figure 3:**
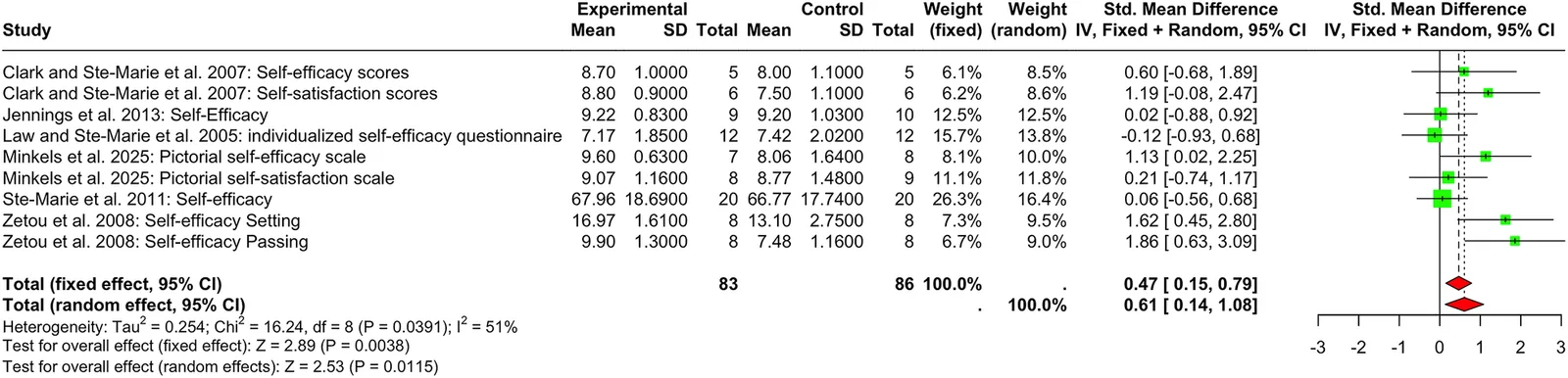
Forest Plot of Effect Eizes Comparing Self-modeling with Control in the Self-efficacy Meta-Analysis.

In the self-efficacy meta-analysis, nine effect sizes from six studies were included to determine the overall impact of self-modeling on self-efficacy. The results indicated that, compared with the control group, self-modeling had a moderately positive overall effect on self-efficacy [SMD = 0.62; 95% CI (0.14, 1.08); *p* = 0.012], with significant heterogeneity across studies (*τ*^2^ = 0.254; *I*^2^ = 51%; *p* = 0.039; Figure 3). Regression analysis revealed that the effect of self-modeling on self-efficacy was moderated by the total duration and frequency of interventions, but not by age (Table 3). These findings indicate that the dose of the intervention is a significant moderator of self-efficacy enhancement. This result is consistent with the conclusions of meta-analyses in the field of athletic performance, further confirming the dose-dependent nature of the self-modeling effect. Furthermore, the self-modeling effect demonstrates good consistency in enhancing self-efficacy across different age groups.

**Table 3:** Meta-regression analyses of the self-efficacy outcome data synthesized in the self-efficacy meta-analysis.

Subgroup	*N*	*K*	Beta	*P*	Sig.	*τ* ^2^	*I* ^2^
Total time	6	9	0.0018	<0.01	Yes	0	0
Session number	6	9	0.124	<0.01	Yes	0	0
Age	6	9	−0.034	0.71	No	0.35	59.1

*N*, number of studies; *K*, number of effect sizes; SMD, standardized mean difference; 95% CI: lower and upper confidence intervals; *P*, significance value; *τ*^2^, measure of variation around average; *I*^2^, measure of proportion of observed variance (%); Sig. mod., comparison between subgroup categories; NA, insufficient number of data points for the analysis; ref, reference category; ns, no significant difference compared with reference category.

### 3.6 Sensitivity analysis

Sensitivity analyses were performed by excluding outliers (Supplementary Table S6). For the movement meta-analysis, results indicating a significant positive effect of self-modeling on motor outcomes compared with control conditions were reliable. A sensitivity analysis of the follow-up data revealed that, 1 week after the intervention, the comparison between the self-modeling and control conditions was not robust. After excluding the study by Zetou et al., the positive effect of self-modeling on motor outcomes remained insignificant (*p* = 0.47).

Sensitivity analysis revealed that there were no outliers in self-efficacy, indicating that individual large or small effect sizes did not have a significant impact on the pooled effect.

### 3.7 Risk of bias and GRADE

**Figure 4:**
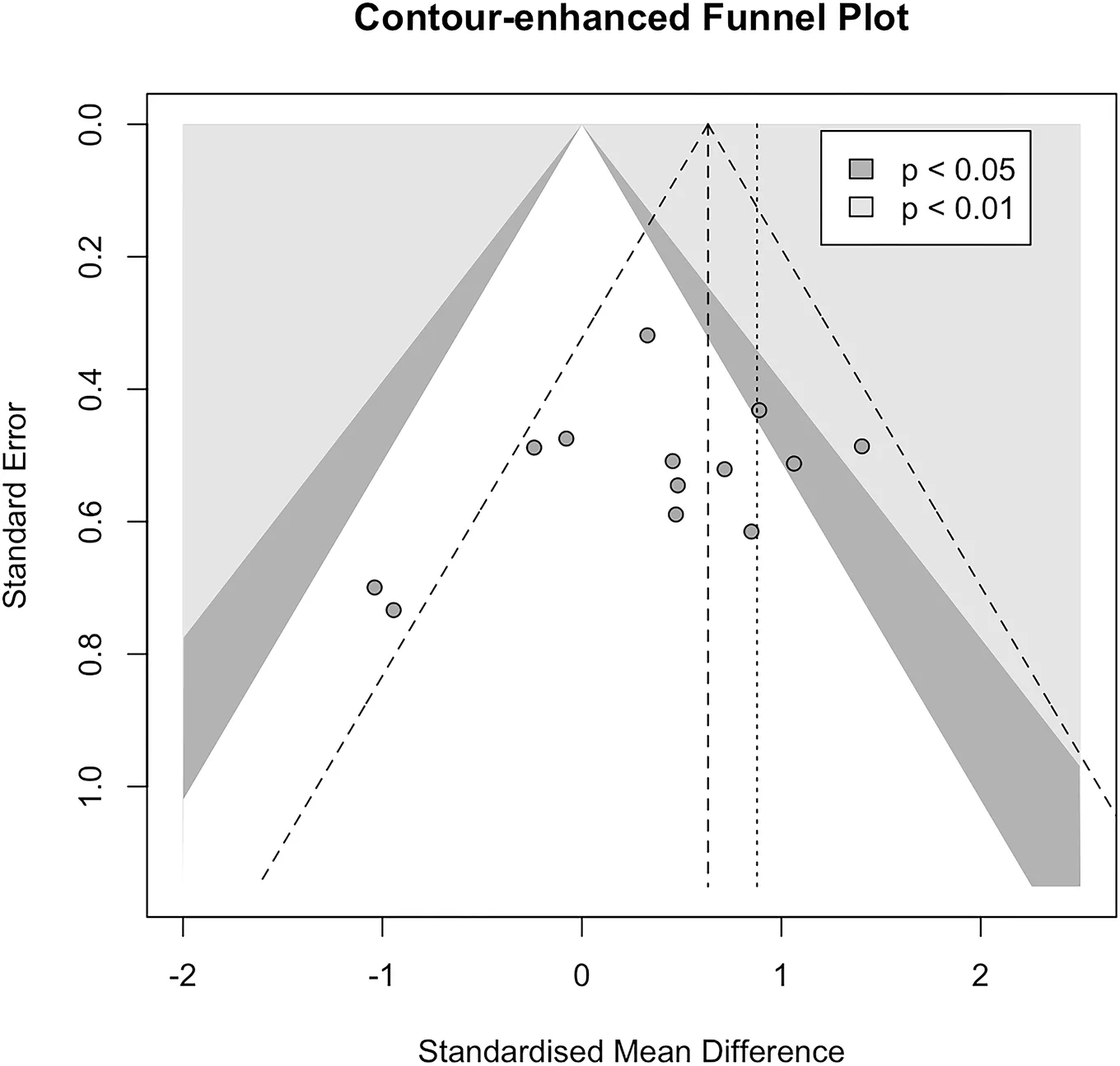
Contour-Enhanced Funnel Plot Comparing Self-modeling with the Control Condition in the Movement Meta-Analysis.

In the meta-analysis of exercise, when comparing the self-modeling group with the control group, funnel plot inspection (Figure 4) and Egger's intercept test (Egger's *β*₀ = 3.14, *p* > 0.01) indicated a low likelihood of publication bias.

**Figure 5:**
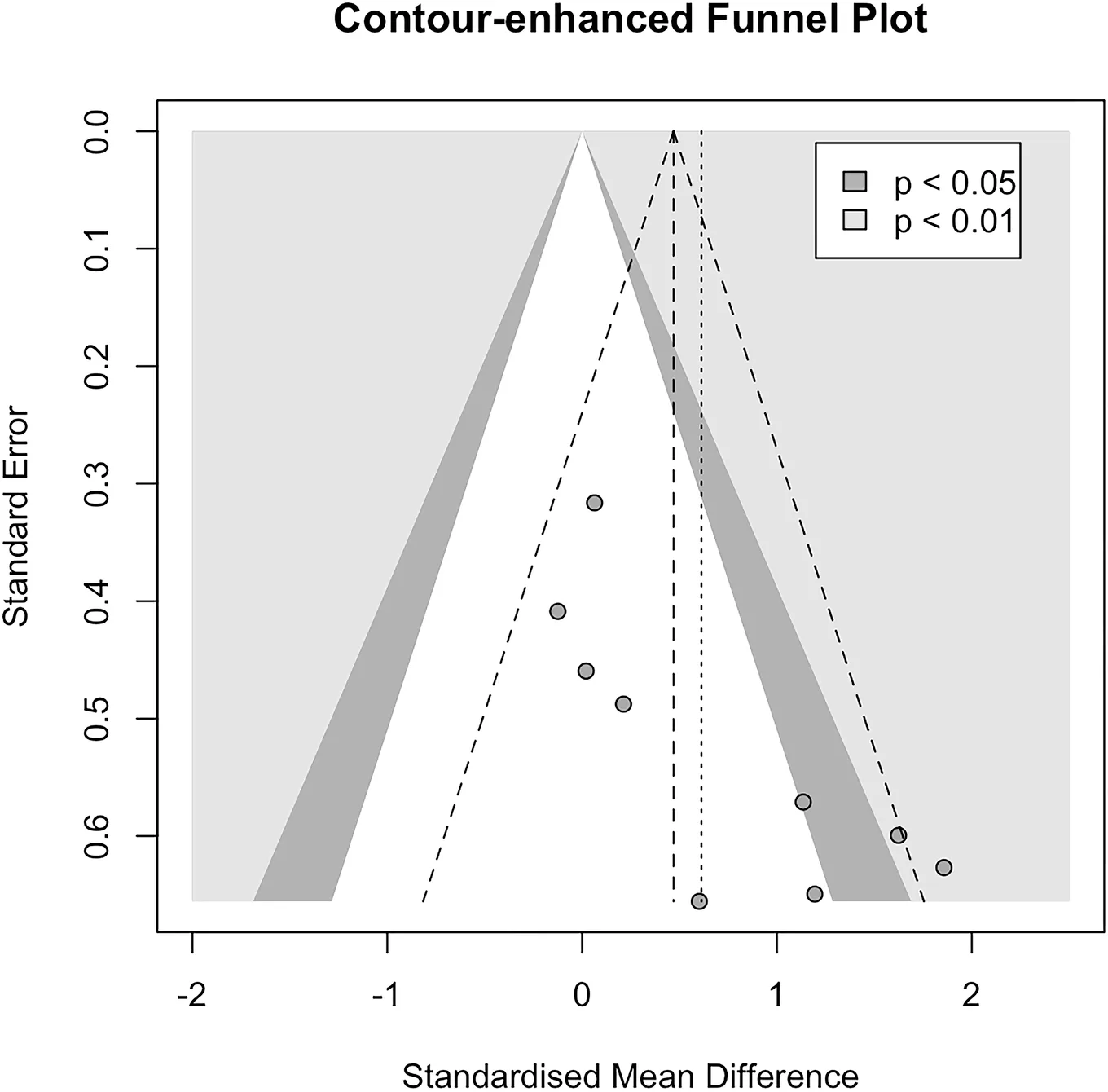
Contour-Enhanced Funnel Plot Comparing Self-modeling with the Control Condition in the Self-efficacy Meta-Analysis.

In the self-efficacy meta-analysis, funnel plot inspection (Figure 5) and Egger's intercept test (Egger's *β*₀ = 4.28 with *p* > 0.01) indicated no statistically significant publication bias. However, the interpretation of this result should be treated with caution due to the limited number of included studies.

## 4 Discussion

To evaluate the impacts of self-modeling on athletic performance and self-efficacy, we carried out two meta-analyses. The results of the movement meta-analysis indicated that self-modeling had a positive effect on overall exercise outcomes, ranging from small to large, with these improvements dissipating within 1 week after the intervention ended. The results of the meta-analysis on self-efficacy indicated that self-modeling produced a moderately positive effect on self-efficacy. These findings confirm that self-modeling is an effective intervention for enhancing athletic performance and self-efficacy.

### 4.1 Movement meta-analysis

The movement meta-analysis indicates that self-modeling significantly improves movement performance in a training environment. This positive effect is evident in most of the included studies spanning sports such as swimming (11, 16, 36), running (35), and figure skating (40). This suggests that self-modeling may be more beneficial for sports that focus on movement economy and performance (62). This is likely due to self-modeling's ability to provide multidimensional movement information, particularly visual feedback regarding spatial, temporal, and coordination aspects (41–43). Dynamic, intuitive self-visual feedback conveys the temporal relationships, spatial trajectories, and overall coordination patterns among different body segments during complex movements more effectively than traditional verbal instruction or external demonstrations (43). Traditional methods often fall short when conveying such multidimensional, synchronous tacit knowledge. They are constrained by the limitations of linguistic description alone or the gap between external perspectives and one's own perceptions, resulting in an indirect and incomplete representation (39). For instance, in sports involving sequential coordinated movements such as volleyball serves and freestyle swimming, learners can more clearly identify subtle differences in movement trajectories, rhythms, and body postures by watching videos of their own peak performances. This enables them to construct more refined motor representations at the cognitive level (36). This representation enhances the psychological simulation of movements and may promote the adaptive reorganization of the neuromuscular system (44). This enables more economical and efficient execution of movements (16). However, it is worth noting that this positive impact may be most evident as an immediate effect. The meta-analysis of follow-up data collected 1 week after the intervention revealed that the positive effects of self-modeling were not sustained. This suggests that the fine motor representations or neuromuscular adaptations induced by watching self-modeling videos will diminish over time if not reinforced by continuous visual feedback or targeted practice (45). One possible explanation is that the long-term consolidation of motor skills requires repeated retrieval and reinforcement. Although single or short-term self-modeling interventions can optimize cognitive representations during the initial learning phase, these interventions are insufficient for supporting long-term skill retention (36). Furthermore, regression analysis reveals that the total duration and frequency of the intervention can moderate the effects of self-modeling. Intervention designs included in the study varied, encompassing both acute and long-term interventions. Regardless of the intervention model employed, there was an overall trend indicating that the more intensive the intervention, the more pronounced the improvement in athletic performance. This finding further supports the dose–response hypothesis, which implies that a sufficient and sustained intervention may be necessary to maintain the effects of self-modeling.

Regarding the methodological moderators that may influence self-modeling, we found statistically significant positive correlations between total intervention duration and effect size, and between intervention frequency and effect size. This indicates that achieving better outcomes may necessitate sufficient and sustained intervention. One possible explanation is that learning and consolidating motor skills are inherently neurocognitive processes that require repeated reinforcement (46, 47). Longer total duration and increased viewing frequency provide learners with more opportunities to consolidate action representations established through video and integrate them into long-term memory (48–50). This translates into more stable behavioral change (36). Apart from the intervention dose, no substantial correlations were identified between the age of the participants, athletic experience, the skill classification, or the control group intervention type and the effect size. The failure of skill classification to emerge as a key moderating variable is worthy of consideration. Previous research has suggested that continuous skills may benefit more from continuous visual feedback than discrete skills (39). However, the results of the meta-analysis suggest that this advantage is not statistically robust. This implies that learners of different skill levels could extract valuable information from observing their own successful performances. Differences may be obscured by substantial heterogeneity in task specificity, measurement sensitivity, and video-editing quality across studies (16). The lack of significant moderation by age implies that self-modeling effectively provides intuitive, self-referential information through its fundamental cognitive mechanisms in both children and adults (36). Furthermore, control group intervention type did not have a moderating effect. This indicates that the effects of self-modeling may not be solely due to the design of the control group but rather due to the inherent advantages of the intervention method itself.

In general, the movement meta-analysis interventions show that self-modeling has the potential to be an effective strategy. The intervention dose is currently a well-defined moderating factor, while the moderating effects of other demographic or task-related variables remain unclear. To more precisely define the optimal application scenarios for self-modeling, future research should explore the potential interactions among these factors.

### 4.2 Self-efficacy meta-analysis

The results of the self-efficacy meta-analysis indicate that self-modeling has a statistically significant positive effect on enhancing individuals' self-efficacy compared with control conditions, with a medium effect size. This finding is consistent with the results of several empirical studies showing that individuals can boost their confidence by watching videos of themselves performing skills successfully (12, 38). The underlying mechanism may be that self-modeling provides a visual representation of “mastering experiences” and reinforces positive perceptions of learners' abilities through repeated observation of successful performances in videos (1, 7).

The results of the moderator variable analysis indicate that the intervention dose has a significant effect on the outcomes of self-modeling. The greater the intervention dose, the greater the enhancement of self-efficacy (51–53). This may be because more frequent or prolonged video observation provides learners with more opportunities for self-observation, self-comparison, and self-regulation (54–57). This gradually helps them internalize successful performance patterns (9, 58). However, some studies suggest that the dose–response effect is not always applicable. In particular, increasing the frequency of interventions may lead to diminishing returns when athletes already possess a high baseline level of self-efficacy or skill proficiency (40). Future research should explore the applicability of self-modeling at different experience levels. Employing more refined mediation or moderation models, such as integrating self-regulation theory (59) or attention control mechanisms, could elucidate the specific psychological and behavioral pathways through which self-modeling influences self-efficacy.

The age variable did not reveal a significant moderating effect. This implies that the promotional effect of self-modeling on self-efficacy is similar across age groups. Thus, self-modeling appears to be a universally applicable visual cognitive intervention (60, 61). However, the insignificant age effects may be related to the relatively narrow age range of the samples selected for the studies. For example, many studies have focused on adolescent athletes (38, 40). Future research should examine a broader age range to validate this conclusion.

It is worth noting that the self-efficacy meta-analysis did not include subgroup analyses for age, skill classification, or athletic experience. Therefore, it is not possible to thoroughly examine the differential effects of self-modeling across varying levels of experience or in specific sports contexts. For example, research indicates that novice athletes may rely more heavily on external video feedback due to weaker self-regulation abilities, while elite athletes may be better at extracting technical details from self-modeling rather than deriving self-efficacy information (40).

In summary, self-modeling exerts a positive effect on self-efficacy, which is significantly moderated by intervention dose but independent of age.

### 4.3 Limitations

This review synthesizes comprehensive evidence regarding the effects of self-modeling on athletic performance and self-efficacy. Nevertheless, several limitations exist. One issue is the small number of studies available for the self-efficacy outcome. This prevented us from running subgroup analyses and limited our ability to explore methodological moderators. The small study pool also weakened statistical power. Therefore, we urge caution when interpreting the meta-analytic estimates. We also lack sufficient data on long-term effects. Most primary studies did not include extended follow-up assessments. This means we cannot determine with confidence whether the benefits of self-modeling last over time. Another limitation concerns the age range of the sample. Most participants were adolescents. This narrow coverage restricts how well we can apply our findings to younger children, older adults, or other populations. In addition, publication bias warrants consideration in this review. Egger's test and funnel plots showed no clear asymmetry. However, these methods have low reliability when fewer than 10 studies are included. Thus, we cannot fully rule out the possibility of small-study effects or unpublished null results. Future research with larger, well-designed trials will help confirm our conclusions and refine how best to deliver self-modeling interventions.

## 5 Conclusion

This study systematically evaluated the effects of self-modeling on athletic performance and self-efficacy through two meta-analyses. The results demonstrated that self-modeling significantly and positively impacted athletic performance and self-efficacy. However, 1 week after the intervention ended, the improvement in athletic outcomes attributed to self-modeling was no longer statistically significant. Concurrently, the dosage of the intervention was the primary factor influencing postintervention outcomes in both meta-analyses. There was a quantitative relationship between the dosage of the intervention and the improvement effects, with greater dosages yielding more pronounced effects. Furthermore, skill type, age, and athletic experience did not significantly moderate the effect of the self-modeling intervention on exercise outcomes, indicating that this approach possesses broad applicability across diverse populations. Future research should focus on identifying the optimal dosage of self-modeling interventions, examining their effectiveness in formal competitive settings, clarifying the moderating effects of task characteristics, skill classification, and athletic experience on self-efficacy, and understanding the pathways through which they influence self-efficacy. This will optimize intervention protocols and extend the durability of the benefits.

## Data Availability

The original contributions presented in the study are included in the article/Supplementary Material, further inquiries can be directed to the corresponding author.
