# Supplementary material for: The impact of self-modeling on athletic performance and self-efficacy: a systematic review and meta-analysis
Source: Front Sports Act Living. 2026 Aug 19;8:1809566. doi: 10.3389/fspor.2026.1809566 (PMC13533993; doi:10.3389/fspor.2026.1809566)
Supplement: Supplementary file 1 [file Table1.docx]

Supplementary Material

Table S1: Search Sources and Corresponding Results

| **Data source** | **Search query** | **Results** |
| --- | --- | --- |
| **Web of Science** | ALL=(self-modeling or self-referential modeling) AND ALL=((sport or exercise or workout or physical activity or movement) OR ALL=(self-efficacy or perceived self-efficacy or self-efficacy belief)) | 160 |
| **PubMed** | (self-modeling[Title/Abstract] OR self-referential modeling[Title/ Abstract]) AND ((sport[Title/Abstract] OR exercise[Title/Abstract] OR workout[Title/Abstract] OR physical activity[Title/Abstract] OR movement[Title/Abstract]) OR (self-efficacy[Title/Abstract] OR perceived self-efficacy[Title/Abstract] OR self-efficacy belief[Title/Abstract])) | 57 |
| **MEDLINE** | (self-modeling OR self-referential modeling) AND ((sport OR exercise OR workout OR physical activity OR movement) OR (self-efficacy OR perceived self-efficacy OR self-efficacy belief)) | 70 |
| **SPORTDiscus** | (self-modeling OR self-referential modeling) AND ((sport OR exercise OR workout OR physical activity OR movement) OR (self-efficacy OR perceived self-efficacy OR self-efficacy belief)) | 73 |
| **Scopus** | (TITLE-ABS-KEY (self-modeling OR self-referential modeling) AND TITLE-ABS-KEY ((sport OR exercise OR workout OR physical activity OR movement) OR (self-efficacy OR perceived self-efficacy OR self-efficacy belief))) | 28 |
| **PsycINFO** | (self-modeling OR self-referential modeling) AND ((sport OR exercise OR workout OR physical activity OR movement) OR (self-efficacy OR perceived self-efficacy OR self-efficacy belief)) | 52 |

Table S2: Characteristics of Studies Included in the Movement Meta-Analysis

| **study** | **Sample Size** | **Age (mean+SD)** | **Sex (men/**  **women)** | **Interventions** | **Duration** | **Frequency** | **Session Number** | **Session Duration** | **Outcome Measures** |
| --- | --- | --- | --- | --- | --- | --- | --- | --- | --- |
| Law and Ste-Marie et al.  2005 | SM (12) | 13.4 (1.4) | 0/12 | Self-modeling + physical training | 3 weeks | 2 times/week | 6 | 15 min | Performance Outcome |
|  | CG (12) |  |  | Physical training |  |  |  |  | Performance Form |
| Barzouka et al.  2007 | SM (16) | 13.1 (0.9) | 0/16 | Self-modeling + verbal guidance + physical training | 6 weeks | 2 times/week | 12 | 45 min | Reception performance outcome  Reception technique |
|  | CG (19) |  | 0/19 | Verbal guidance + physical training |  |  |  |  |  |
| Clark and Ste-Marie et al.  2007 | SM (11) | 8.3 (1.2) | 4/7 | Self-modeling + physical training | 6 days | 1 time/day | 6 | 30 min | Swimming performance |
|  | CG (11) |  | 5/6 | Physical training |  |  |  |  |  |
| Zetou et al.  2008 | SM (16) | 12.8 (0.53) | 0/32 | Self-modeling + verbal guidance | 8 weeks | 2 times/week | 16 | 1 h | Setting  Passing |
|  | CG (16) |  |  | Verbal guidance |  |  |  |  |  |
| Ste-Marie et al.  2011 | SM (20) | 12.18 (2.5) | 0/20 | Self-modeling | Unclear | Unclear | 2 | 1-1.5 min | Physical performance |
|  | CG (20) |  |  | No intervention |  |  |  |  |  |
| Jennings et al.  2013 | SM (9)  CG (10) | 13.2 (2.1)  13.9 (0.9) | 12/7 | Self-modeling + verbal guidance + physical training | 2 weeks | 2 times/week | 4 | 1 h | 60 m standing start time  Coach Rating |
|  |  |  |  | Verbal guidance + physical training |  |  |  |  |  |
|  | VSM (12)  CG (12) | 26.8 (6) |  | Self-modeling + running  Running | 1 day | 1 time/day | 1 | Unclear | Time to Exhaustion |
| Hagin et al.  2015 |  |  |  |  |  |  |  |  |  |
|  |  |  | 12/0 |  |  |  |  |  |  |
|  |  |  |  |  |  |  |  |  |  |
|  | SM-VB (9)  CG (9) |  |  |  |  |  |  |  |  |
| Gonzales et al.  2015 |  | 22.6 (2.1) | 9/0 | Front-facing self-modeling video + running |  |  |  |  |  |
|  |  |  |  |  | 1 day | 1 time/day | 1 | Unclear | Time to Exhaustion |
|  |  |  |  | Running |  |  |  |  |  |
| Giannousi et al.  2017 | SM (16) | 18.7 (1.82) | 16/0 | Self-modeling + verbal guidance | 7 weeks | 1 time/week | 7 | 40 min | Hands technique scores in freestyle swimming  Time of the 25-meter freestyle swim |
|  | CG (15) |  | 15/0 | Verbal guidance |  |  |  |  |  |
| Minkels et al.  2025 | SM (15)  CG (17) | 6.29 (1.11)  6.76 (1.51) | 6/9 | Self-modeling + physical training + feedback | 6 weeks | 1 time/week | 6 | 1 h | Swimming Time  Modified front crawl rating scale |
|  |  |  | 7/10 | Physical training + feedback |  |  |  |  |  |

Table S3: Characteristics of Studies Included in the Self-efficacy Meta-Analysis

| **study** | **Sample Size** | **Age (mean + SD)** | **Sex (men/women)** | **Interventions** | **Duration** | **Frequency** | **Session Number** | **Session Duration** | **Outcome Measures** |
| --- | --- | --- | --- | --- | --- | --- | --- | --- | --- |
| Law and Ste-Marie et al. 2005 | SM (12) | 13.4 (1.4) | 0/12 | Self-modeling + physical training | 3 weeks | 2 times/week | 6 | 15 min | Individualized self-efficacy questionnaire |
|  | CG (12) |  |  | Physical training |  |  |  |  |  |
| Clark and Ste-Marie et al. 2007 | SM (11) | 8.3 (1.2) | 4/7 | Self-modeling + physical training | 6 days | 1 time/day | 6 | 30 min | Self-efficacy scores  Self-satisfaction scores |
|  | CG (11) | 8.3 (1.2) | 5/6 | Physical training |  |  |  |  |  |
| Zetou et al.  2008 | SM (16) | 12.8 (0.53) | 0/32 | Self-modeling + verbal guidance | 8 weeks | 2 times/week | 16 | 1 h | Self-efficacy Setting  Self-efficacy Passing |
|  | CG (16) |  |  | Verbal guidance |  |  |  |  |  |
| Ste-Marie et al. 2011 | SM (20) | 12.18 (2.5) | 0/20 | Self-modeling  No intervention |  |  |  |  |  |
|  |  |  |  |  | Unclear | Unclear | 2 | 1-1.5 min | Self-efficacy |
|  | CG (20) |  |  |  |  |  |  |  |  |
| Jennings et al. 2013 | SM (9)  CG (10) | 13.2 (2.1)  13.9 (0.9) | 12/7 | Self-modeling + verbal guidance + physical training | 2 weeks | 2 times/week | 4 | 1 h | Self-Efficacy |
|  |  |  |  | Verbal guidance + physical training |  |  |  |  |  |
| Minkels et al. 2025 | SM (15)  CG (17) | 6.29 (1.11)  6.76 (1.51) | 6/9  7/10 | Self-modeling + physical training + feedback | 6 weeks | 1 time/week | 6 | 1 h | Pictorial self-efficacy scale  Pictorial self-satisfaction scale |
|  |  |  |  | Physical training + feedback |  |  |  |  |  |

Table S4: Quality Assessment of Studies Included in the Movement Meta-Analysis

| **Study** | **Eligibility criteria** | **Random allocation** | **Concealed allocation** | **Similar baseline** | **Participant blinding** | **Investigator blinding** | **Assessor blinding** | **Completeness of follow-up** | **Intention to treat** | **Between group comparisons** | **Point measures and variability** | **Total Score** | **Overall quality** |
| --- | --- | --- | --- | --- | --- | --- | --- | --- | --- | --- | --- | --- | --- |
| Jennings et al. 2013 | 1 | 1 | 0 | 1 | 0 | 0 | 0 | 1 | 1 | 1 | 1 | 7 | High |
| Barzouka et al. 2007 | 1 | 1 | 0 | 0 | 0 | 0 | 0 | 1 | 1 | 1 | 1 | 6 | High |
| Clark and Ste-Marie et al. 2007 | 1 | 1 | 0 | 1 | 0 | 0 | 1 | 1 | 1 | 1 | 1 | 8 | High |
| Giannousi et al. 2017 | 1 | 1 | 0 | 1 | 0 | 0 | 1 | 1 | 1 | 1 | 1 | 8 | High |
| Law and Ste-Marie et al. 2005 | 1 | 0 | 0 | 1 | 0 | 0 | 1 | 1 | 1 | 1 | 1 | 7 | High |
| Minkels et al. 2025 | 1 | 1 | 0 | 1 | 0 | 0 | 1 | 1 | 0 | 1 | 1 | 7 | High |
| Ste-Marie et al. 2011 | 1 | 1 | 0 | 0 | 0 | 0 | 0 | 1 | 1 | 1 | 1 | 6 | High |
| Zetou et al. 2008 | 0 | 1 | 0 | 1 | 0 | 0 | 0 | 1 | 0 | 1 | 1 | 5 | Medium |
| Hagin et al. 2015 | 1 | 1 | 0 | 1 | 0 | 0 | 0 | 1 | 1 | 1 | 1 | 7 | High |
| Gonzales et al. 2015 | 1 | 1 | 0 | 1 | 0 | 0 | 0 | 1 | 1 | 1 | 1 | 7 | High |
| Total | 9 | 9 | 0 | 8 | 0 | 0 | 4 | 10 | 8 | 10 | 10 | 6.8 | High |

Table S5: Quality Assessment of Studies Included in the Self-efficacy Meta-Analysis

| **Study** | **Eligibility criteria** | **Random allocation** | **Concealed allocation** | **Similar baseline** | **Participant blinding** | **Investigator blinding** | **Assessor blinding** | **Completeness of follow-up** | **Intention to treat** | **Between group comparisons** | **Point measures and variability** | **Total Score** | **Overall quality** |
| --- | --- | --- | --- | --- | --- | --- | --- | --- | --- | --- | --- | --- | --- |
| Jennings et al. 2013 | 1 | 1 | 0 | 1 | 0 | 0 | 0 | 1 | 1 | 1 | 1 | 7 | High |
| Clark and Ste-Marie et al. 2007 | 1 | 1 | 0 | 1 | 0 | 0 | 1 | 1 | 1 | 1 | 1 | 8 | High |
| Law and Ste-Marie et al. 2005 | 1 | 0 | 0 | 1 | 0 | 0 | 1 | 1 | 1 | 1 | 1 | 7 | High |
| Minkels et al. 2025 | 1 | 1 | 0 | 1 | 0 | 0 | 1 | 1 | 0 | 1 | 1 | 7 | High |
| Ste-Marie et al. 2011 | 1 | 1 | 0 | 0 | 0 | 0 | 0 | 1 | 1 | 1 | 1 | 6 | High |
| Zetou et al. 2008 | 0 | 1 | 0 | 1 | 0 | 0 | 0 | 1 | 0 | 1 | 1 | 5 | Medium |
| Total | 5 | 5 | 0 | 5 | 0 | 0 | 3 | 6 | 4 | 6 | 6 | 6.67 | High |

Table S6: Pooled effect size after excluding outliers in the movement meta-analysis

| Groups | Research from | Exclusion effect size | SMD  (95% CI) | P | I^2^ |
| --- | --- | --- | --- | --- | --- |
| Self-modeling vs CG | Giannousi et al. 2017 | Hands technique scores in freestyle swimming | 0.49  (0.21, 0.77) | 0.0005 | 21 |
|  | Jennings et al. 2013 | Coach Rating |  |  |  |
|  | Zetou et al. 2008 | Setting |  |  |  |
|  |  | Passing |  |  |  |

Table S6: Pooled effect size after excluding outliers in the movement follow-up meta-analysis

| Groups | Research from | Exclusion effect size | SMD  (95% CI) | P | I^2^ |
| --- | --- | --- | --- | --- | --- |
| Self-modeling vs CG | Zetou et al. 2008 | Setting | 0.14  (-0.24, 0.52) | 0.47 | 0 |
|  |  | Passing |  |  |  |

Table S7: Quality Assessment Form for Studies Included in the Movement Meta-Analysis

| **Certainty assessment** | | | | | | | **Effect** | | **Certainty** | **Importance** |
| --- | --- | --- | --- | --- | --- | --- | --- | --- | --- | --- |
| **№ of studies** | **Study design** | **Risk of bias** | **Inconsistency** | **Indirectness** | **Imprecision** | **Other considerations** | **Relative (95% CI)** | **Absolute (95% CI)** |  |  |
| 10 | randomised trials | serious^a^ | serious^b^ | not serious | not serious | none | - | SMD 0.93  SD higher (0.34 higher to 1.52 higher) | ⨁⨁◯◯ Low^a^ | CRITICAL |

**CI**: confidence interval; **SMD**: standardised mean difference; **a**: Bias of Blinding; **b**: Rating Bias.

Table S8: Quality Assessment Form for Studies Included in the Self-efficacy Meta-Analysis

| **Certainty assessment** | | | | | | | **Effect** | | **Certainty** | **Importance** |
| --- | --- | --- | --- | --- | --- | --- | --- | --- | --- | --- |
| **№ of studies** | **Study design** | **Risk of bias** | **Inconsistency** | **Indirectness** | **Imprecision** | **Other considerations** | **Relative (95% CI)** | **Absolute (95% CI)** |  |  |
| 6 | randomised trials | serious^a^ | serious^b^ | not serious | not serious | none | - | SMD 0.54  SD higher (0.14 higher to 0.94 higher) | ⨁⨁◯◯ Low | CRITICAL |

**CI**: confidence interval; **SMD**: standardised mean difference; **a**: Bias of Blinding; **b**: Rating Bias.
